# Comparative Analysis of Serum Amino Acid Profiles in Patients with Myasthenia Gravis and Multiple Sclerosis

**DOI:** 10.3390/jcm13144083

**Published:** 2024-07-12

**Authors:** Piotr Kośliński, Łukasz Rzepiński, Marcin Koba, Zdzisław Maciejek, Mariusz Kowalewski, Emilia Daghir-Wojtkowiak

**Affiliations:** 1 Department of Toxicology and Bromatology, Collegium Medicum in Bydgoszcz, Nicolaus Copernicus University in Toruń, Dr. A. Jurasza 2, 85-089 Bydgoszcz, Poland; kobamar@cm.umk.pl; 2 Department of Neurology, 10th Military Research Hospital and Polyclinic, Powstańców Warszawy 5, 85-681 Bydgoszcz, Poland; luk.rzepinski@gmail.com (Ł.R.); z.maciejek@wp.pl (Z.M.); 3 Sanitas—Neurology Outpatient Clinic, 85-010 Bydgoszcz, Poland; 4 Multiple Sclerosis Foundation, 78-449 Borne Sulinowo, Poland; 5 International Centre for Cancer Vaccine Science, University of Gdansk, 80-309 Gdansk, Poland; emilia.daghir-wojtkowiak@ug.edu.pl

**Keywords:** myasthenia gravis, multiple sclerosis, amino acids, biomarkers

## Abstract

**Background**: Multiple sclerosis (MS) and myasthenia gravis (MG) are autoimmune diseases that attack the central nervous system (CNS) and the neuromuscular junction, respectively. As the common pathogenesis of both diseases is associated with an autoimmune background and the involvement of T and B lymphocytes, the overlapping of selected clinical symptoms may cause difficulties in the differential diagnosis of both diseases. **Methods**: The aim of the study was to use Liquid Chromatography–Electrospray Ionization–Mass Spectrometry (LC–ESI–MS/MS) in conjunction with multivariate statistical analyses to examine the changes in amino acid metabolic profiles between patients with MG, MS, and a control group. **Results**: Comparative analysis of amino acids (AA) between patients with MG, MS, and within the control group allowed for the identification of statistically significant differences in the amino acid profile. Comparing the patients (patients with MS and MG) with the control group, and after taking the results of multiple tests into account, it was observed that amino acids such as ARG, PRO, TRP, CIT were significantly different between the groups. When considering the comparison between the AA concentrations in MS and MG patients, we found three AAs that were significantly different in the MS and MG groups, after correcting for multiple testing (CIT, GABA, and AAA). Higher concentrations of amino acids that showed significant differences were observed in patients with myasthenia gravis. **Conclusions**: Our results have indicated AAs that may prove valuable for improving the diagnostics of MS and MG patients. To better assess the potential utility of these markers, their performance requires further validation in a larger study group and limitation of possible confounding factors, e.g., medications and diet.

## 1. Introduction

Neurological diseases that are based on immunological mechanisms have been studied for years, and many aspects still remain unclear [1]. Autoimmune mechanisms are associated with both myasthenia gravis (MG) and multiple sclerosis (MS). These diseases are characterized by inflammation and immune deregulation. In the case of MG, antibodies are formed against proteins at the postsynaptic neuromuscular junctions (NMJ), which leads to a unique set of clinical symptoms such as variable muscle fatigue and respiratory complications [2]. In MS, the dysfunction of regulatory T cells (Tregs) ultimately leads to an unregulated T cell response against the myelin structures of the central nervous system (CNS) [3]. MG and MS have been found to affect women more than men in terms of prevalence and incidence. The female to male ratio in patients with MG and MS has been described as approximately 2:1 and 3:1, respectively, but varies depending on age and/or type of disease [4,5]. Although the mechanisms underlying impaired self-tolerance in these and other autoimmune diseases have not been fully elucidated, one possibility is numerical, functional, and/or migratory deficits in T regulatory (Treg) cells. Dysregulation of suppressive and migratory markers on Tregs has been linked to the pathogenesis of both MS and MG. It seems that the discovery of a common pathway in the pathogenesis of related autoimmune diseases may guide future methods of their diagnosis and therapy [6].

The application of metabolomics towards understanding the manifestation and progression of complex neurological diseases represents a powerful means to identify the earliest markers associated with disease progression and treatment response [7]. Results of the previous studies focusing on non-targeted and targeted metabolic analysis in MG and MS patients have allowed us to confirm the differences in metabolic profiles between the study and control group. The results obtained by Lu et al. using untargeted serum metabolic analysis allowed for the separation of healthy people from MG patients based on their metabolic profiles [8]. The use of a double control group (a group of healthy people and patients with rheumatoid arthritis) made it possible to distinguish seropositive patients with MG from the reference autoimmune disease, i.e., rheumatoid arthritis, based on metabolic profiles [9]. Rispoli et al., in their review, offered evidence in support of the potential of metabolomics as a biomarker and drug discovery tool in MS [10].

The interest in amino acids (AA) in MS and MG has resulted from their role in the autoimmune process. AAs are important in the functioning of T lymphocytes, and their disturbed metabolism may be associated with the process of autoimmunity [11,12,13]. AA metabolic profiling has many applications including screening, diagnosis, and treatment monitoring. Scientific research is still being developed to assess the possible use of AAs in the diagnosis and monitoring of therapy in neurodegenerative diseases [14], Alzheimer’s disease [15], Parkinson’s disease [16], autoimmune diseases [17], and cancer [18]. The available literature data indicate that the observed abnormalities in AA metabolic pathways may be related to various clinical aspects (i.e., etiology, pathogenesis, diagnosis, prognosis and treatment) of both multiple sclerosis [19,20,21] and myasthenia gravis [9,22].

Our previous research analyzed the profile of AAs in the serum of MS patients and of MG patients, as well as compared them with the control group, taking into account the differences depending on the disease outcomes. The study revealed different patterns of serum AA profiles between the patients and control group, as well as between the patient groups with various disease types [20,22,23].

It is believed that it is very important to include, in the metabolomic analysis, both a comparative analysis of a group of patients with a control group of healthy people, as well as a comparison of the metabolic profiles of patients from the same group of diseases, e.g., autoimmune diseases. This allows for the detection of more specific changes that may accompany specific diseases such as MG or MS. According to the available knowledge, there are no studies allowing for a direct comparison of the AA profile in patients with MG and MS. Therefore, the aim of the presented work is to use HPLC with mass spectrometry detection, combined with multivariate statistical analyses, to compare AA metabolic profiles in patients with MS and MG in order to identify specific changes.

## 2. Materials and Methods

### 2.1. Subjects and Serum Samples

Patients were recruited from the Neurology Outpatient Clinic at Sanitas (Bydgoszcz, Poland) and the MS Rehabilitation Centre (Borne Sulinowo, Poland). The study population was divided into three groups as follows: 28 patients with generalized or ocular subtype of MG, 121 patients with MS, and 53 healthy volunteers with no history of autoimmune diseases (mean age ± SD: 58.38 ± 12.78; 30 men, 23 women). The concentration data of 29 AAs were centered and standardized prior to analysis. The clinical and demographic characteristics of the patients are presented in Table 1.

Blood samples were collected from patients in the morning, following an overnight fast. The collected samples were centrifuged, and the serum was frozen at −80 °C until analysis. AA profiling of the collected samples was performed using the EZ:faast kit. The EZ:faast AA analysis procedure consisted of three sequential steps, i.e., solid-phase extraction, derivatization, and liquid–liquid extraction. The prepared samples were then analyzed using liquid chromatography–mass spectrometry.

### 2.2. Chemicals and Reagents

In the present study, the following reagents were used: HPLC-grade methanol (Merck Darmstadt, Germany), ammonium acetate and formic acid (Sigma-Aldrich Co., St. Louis, MO, USA), and the EZ:faast^TM^ amino acid kit (Phenomenex, Inc., Torrance, CA, USA). Water was prepared using the Milli-Q system (Millipore, Bedford, MA, USA).

Quantitative analysis was performed using the internal standard method. A mixture of three standards was used as internal standards, namely homoarginine (HARG), methionine-D3 (Met-D3), and homophenylalanine (HPHE).

### 2.3. Instrumentation and Conditions

The quantitative and qualitative analyses of samples were performed using the High-Performance Liquid Chromatograph Nexera XR LC-20 AD pump (Shimadzu, Kyoto, Japan) and a Nexera XR SIL-20AC autosampler (Shimadzu, Kyoto, Japan), coupled with a mass spectrometer equipped with an electrospray ion source (ESI), the LCMS-8045 Mass Spectrometer (Shimadzu, Kyoto, Japan). The instrument was controlled, and the recorded data were processed using LabSolutions LCMS Ver.5.6 software.

Chromatographic separation was carried on the EZ:faast AA analysis–mass spectrometry column (250 × 3.0 mm, 4 µm) at a column temperature of 35 °C with the corresponding binary mobile phase. Solvent A was 10 mM ammonium formate in water and solvent B was 10 mM ammonium formate in methanol. The mobile phase flow was 0.25 mL/min and took place in a gradient system of 68% B–83% B in 13 min. The injection volume was 1 µL. The mass spectrometry (MS) data were acquired in positive ion mode. Multiple reacting monitoring was used for quantification by monitoring the ion transition of the AAs.

### 2.4. Statistical Analysis

The Kruskal–Wallis test was used to assess differences between the continuous variables’ distribution (age, AA concentration). Post hoc analysis was further used to explore differences between means while controlling the family error rate. Considering non-normally distributed data, differences between the two groups were evaluated using Mann–Whitney U test statistics. Bonferroni correction was used to adjust for multiple testing. Pearson’s correlation was used to study the linear correlation between AAs. All analyses and figures were formulated with Python [24].

## 3. Results

### 3.1. Comparison of AA Concentration between a Group of Patients (MS + MG Patients) and a Control Group of Healthy People

**Figure 1:**
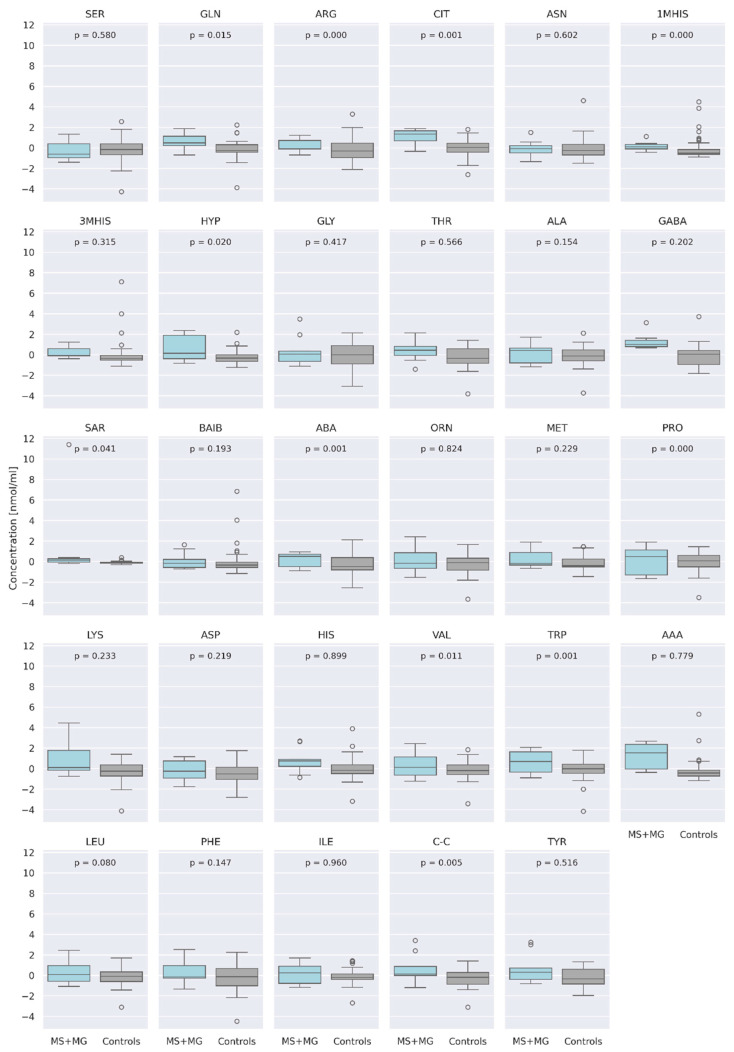
Centered and standardized AA concentrations between two groups—study group (MS and MG (blue) and controls (grey) represented as boxplots. The dots denote outlying observations. Serine (SER), Glutamine (GLN), Arginine (ARG), Citrulline (CIT), Asparagine (ASN), 1-Methyl-L-histidine (1MHIS), 3-Methyl-L-histidine (3MHIS), 4-Hydroxyproline (HYP), Glycine (GLY), Threonine (THR), Alanine (ALA), Gamma-aminobutyric acid (GABA), Sarcosine (SAR), Beta-aminoisobutyric Acid (BAIB), α-Aminobutyric acid (ABA), Ornithine (ORN), Methionine (MET), Proline (PRO), Lysine (LYS), Aspartic acid (ASP), Histidine (HIS), Valine (VAL), Glutamic acid (GLU), Tryptophan (TRP), α-Aminoadipic acid (AAA), Leucine (LEU), Phenylalanine (PHE), Isoleucine (ILE), Cystine (C-C), Tyrsine (TYR).

The concentration data of 29 AA were centered and standardized prior to analysis. In a combined analysis of patients with MS and MG, at a significance level of α = 0.05, we found significant differences in AA concentrations between the study group and the healthy control group for the following amino acids: Glutamine (GLN) (*p* = 0.015), Arginine (ARG) (*p* = 0.0001), Citrulline (CIT) (*p* = 0.0008), 1-Methyl-L-histidine (1MHIS) (*p* = 0.00002), 4-Hydroxyproline (HYP) (*p* = 0.02), Sarcosine (SAR) (*p* = 0.041), α-Aminobutyric acid (ABA) (*p* = 0.0008), Proline (PRO) (*p* = 0.0001), Valine (VAL) (*p* = 0.001), Tryptophan (TRP) (*p* = 0.0006), and Cystine (CC) (*p* = 0.005) (Figure 1). After applying the Bonferroni correction (corrected *p*-value = 0.0017), the concentrations of ARG, CIT, 1MHIS, ABA, PRO, and TRP remained significantly different.

The next step in the analysis involved evaluating AA concentrations as a function of age between the case and control groups. The goal was to determine if there was a trend in AA concentrations related to the age of the study population. Among the patients, no linear trend (positive or negative) was observed with increasing age. However, in the control group, there was a slight negative trend (decrease) in the concentrations of SER, ASN, GLY, GABA, ORN, and ASP, and a slight positive trend (increase) in the concentrations of ARG and CC.

### 3.2. Comparison of AA Concentration between MS and MG Patients

In the next stage of the work, the focus was on comparing the median distribution of AA concentration between the patients with MS and MG. At α = 0.05, we observed a group of AA concentrations, GLN (*p* = 0.015), CIT (*p* = 0.0007), GABA (*p* = 0.00009), SAR (*p* = 0.01), His (*p* = 0.048), and AAA (*p* = 0.0008), to be significantly different between the MS and MG patients.

**Figure 2:**
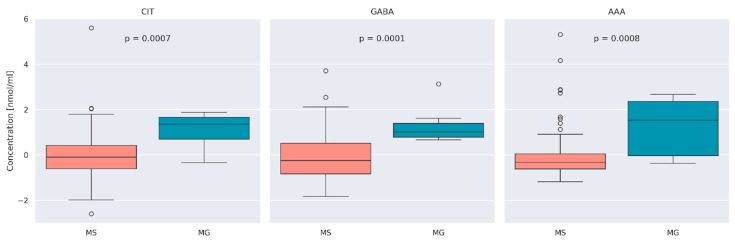
Centered and standardized concentration of Citrulline (CIT)), Gamma-aminobutyric acid (GABA) and α-Aminoadipic acid (AAA) between MS and MG patients. Black dots represent outlying observations.

After Bonferroni correction (corrected *p*-value = 0.0017) concentration of CIT, GABA, and AAA remained significant. The comparison between MS and MG patients in terms of those three AA concentrations is presented as a boxplot (Figure 2).

**Figure 3:**
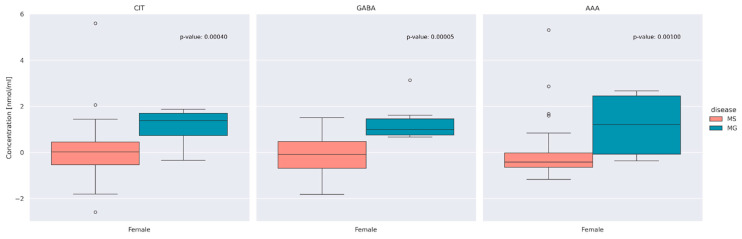
Centered and standardized concentration of Citrulline (CIT), Gamma-aminobutyric acid (GABA) and α-Aminoadipic acid (AAA) between MS and MG patients according to sex. The dots represent outlying observations.

To further explore those differences in the concentrations of CIT, GABA, and AAA between the MS and MG patients, we visualized these concentrations according to sexes (Figure 3). Due to the small number of men in the MG group, only women were included in the comparison.

As can be seen in Figure 3, as well as in the case of unification of the study groups in terms of gender, we observe statistically significantly higher concentrations of CIT, GABA, and AAA in the group of patients with MG.

To further explore the nature of the AA correlations, we examined their relationships with each other. Given that each AA’s distribution approximates a normal distribution, and that the AAs follow similar pathways, a linear relationship between them is anticipated. Therefore, we utilized Pearson’s correlation analysis to assess the degree of linear correlation between the variables.

**Figure 4:**
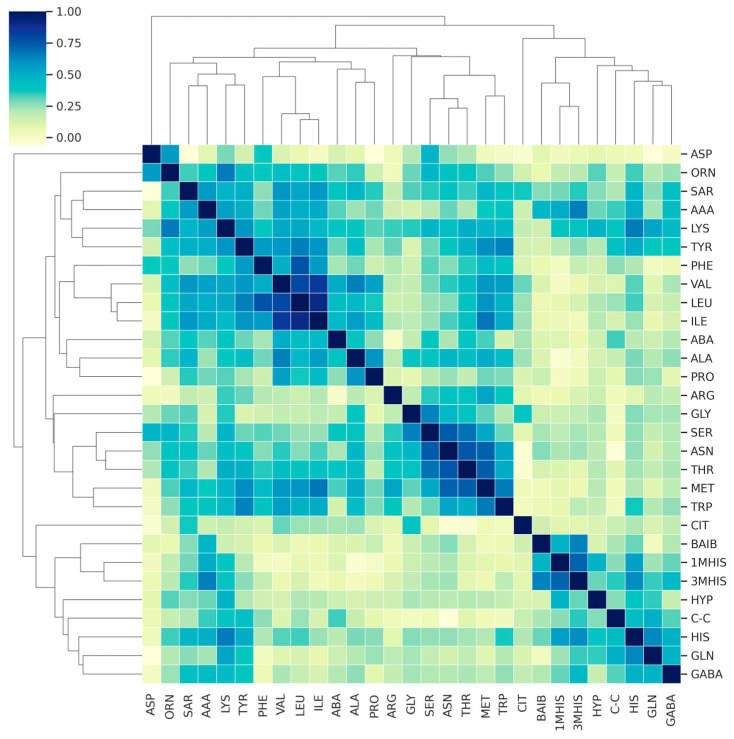
Correlation matrix demonstrating the degree of linear correlation between AAs in the MS and MG patient groups.

**Figure 5:**
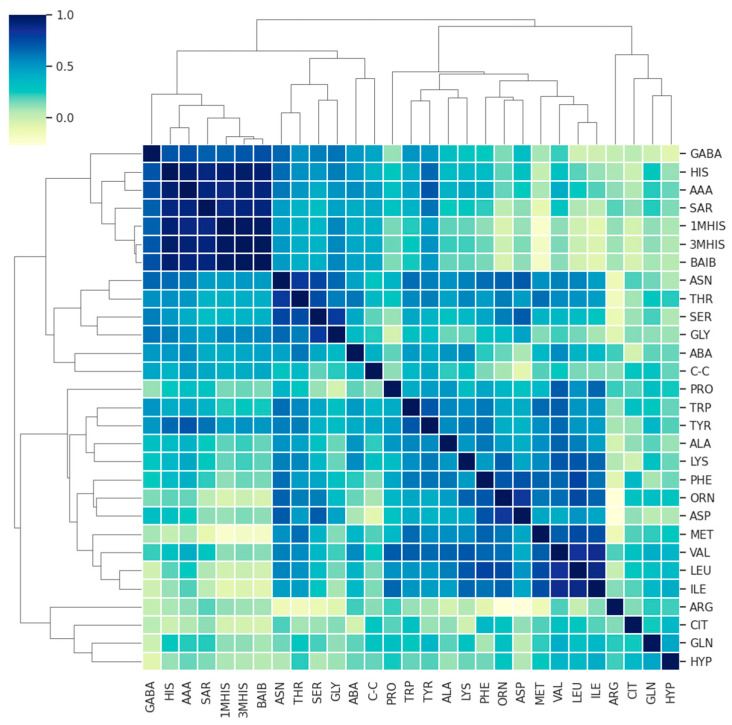
Correlation matrix demonstrating the degree of correlation between AAs in the control group.

Figure 4 presents a correlation matrix showing the degree of linear relationship between the AAs in MS and MG patients, and Figure 5 shows that of the control group. As can be seen from this figure, the vast majority of correlations are weakly positive (a positive trend is expected given that the origin of AA may be driven by a similar mechanism). Several AAs are highly positively correlated with other AAs (R2 > 0.8, dark blue), meaning that they essentially carry the same information.

### 3.3. Evaluation of Differences between Overall AA Concentrations according to the Type of Disease

In the next stage, it was assessed whether there were differences between the general concentrations of AA depending on the type of disease. Patients with MS were divided into three main types of MS, each defined by how far the disease has progressed (RRMS—relapsing–remitting multiple sclerosis; SPMS—secondary progressive multiple sclerosis; PPMS—primary progressive multiple sclerosis). The study included patients with generalized myasthenia gravis (GMG). The Kruskal–Wallis test showed the existence of differences between the disease types in terms of AA concentrations (*p* = 1.04 × 10^−12^). The post hoc analysis revealed the significant differences of AA concentrations between the following:

(i) PPMS (n = 26) and RRMS (n = 41) (*p* = 0.008)
(ii) PPMS (n = 26) and GMG (n = 25) (*p* = 0.03)
(iii) RRMS (n = 41) and GMG (n = 25) (*p* = 0.00001)
(iv) SPMS (n = 55) and GMG (n = 25) (*p* = 0.0008)

**Figure 6:**
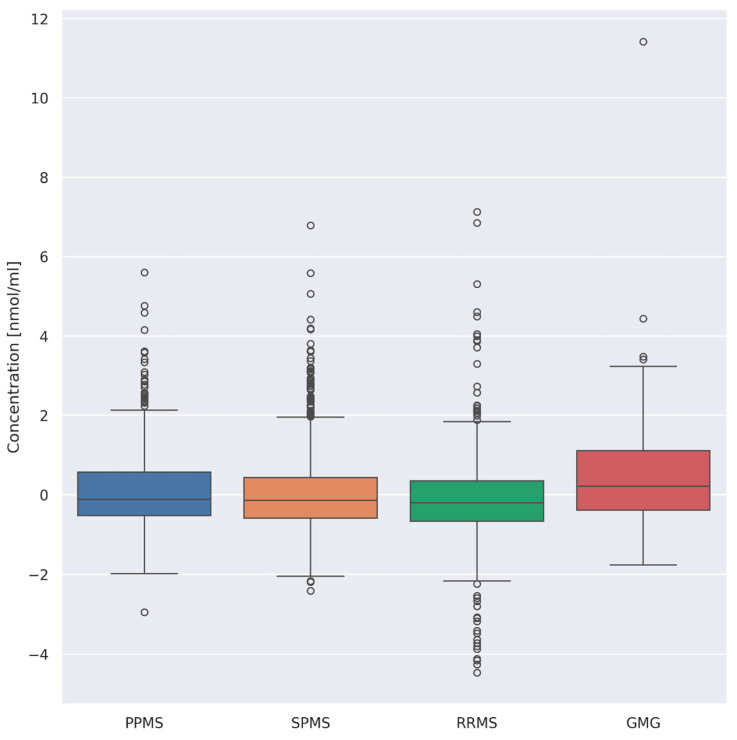
Centered and standardized AA concentrations for each disease type, MS and MG (PPMS—primary progressive multiple sclerosis, SPMS—secondary progressive multiple sclerosis, RRMS—relapsing–remitting multiple sclerosis; GMG—generalized myasthenia gravis).

However, at this point, it should be highlighted that the differences mentioned may be a consequence of the existence of outlying points (Figure 6).

**Figure 7:**
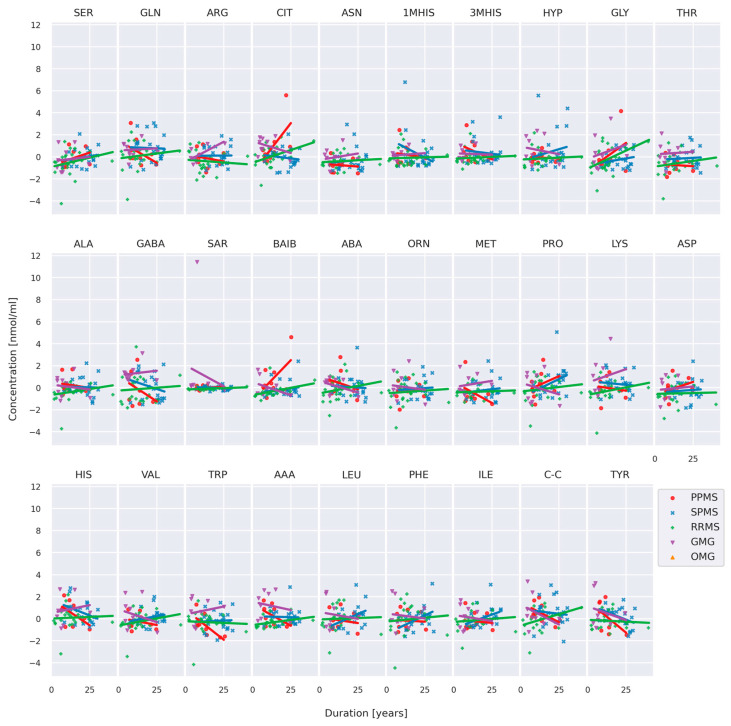
Centered and standardized AA concentration in a function of duration stratified by MS/MG subtypes. (PPMS—primary progressive multiple sclerosis, SPMS—secondary progressive multiple sclerosis, RRMS—relapsing–remitting multiple sclerosis; GMG—generalized myasthenia gravis, OMG—ocular myasthenia gravis).

The trend of AA concentrations was then checked with the duration of MS/MG (patients only) (Figure 7). From visual inspection, there is no trend of AA concentrations with the course of disease duration.

### 3.4. Comparison of AA Concentration between Two Groups of Patients: RRMS (n = 41) and MG (n = 28)

**Figure 8:**
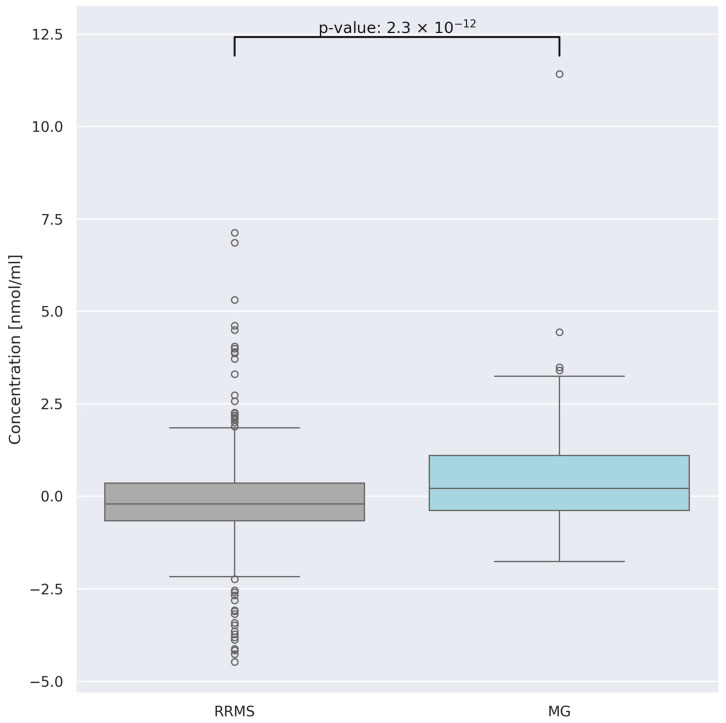
Centered and standardized AA concentrations for two groups: relapsing–remitting multiple sclerosis (RRMS) and myasthenia gravis (MG) patients. The dots represent outlying observations.

Typically, multiple sclerosis (MS) begins with an initial relapsing–remitting course (RRMS). RRMS and myasthenia gravis (MG) share many similarities, including a relapsing course with a wide range of clinical signs, genetic predisposition, and a favorable response to anti-CD20 monoclonal antibodies or other forms of immunosuppressive therapies [25]. Notably, the potential clinical course, characterized by spontaneous or steroid-induced regression of neurological deficits, can complicate the initial differential diagnosis of both diseases. Thus, identifying a serum biomarker that can differentiate between these conditions would be highly beneficial. Consequently, in this study, a group of patients with RRMS was isolated for a comparative analysis with patients with MG. When comparing AA concentrations between the two groups—RRMS (n = 41) and MG (n = 28)—statistically significant differences in total AA concentration were observed (Figure 8).

**Figure 9:**
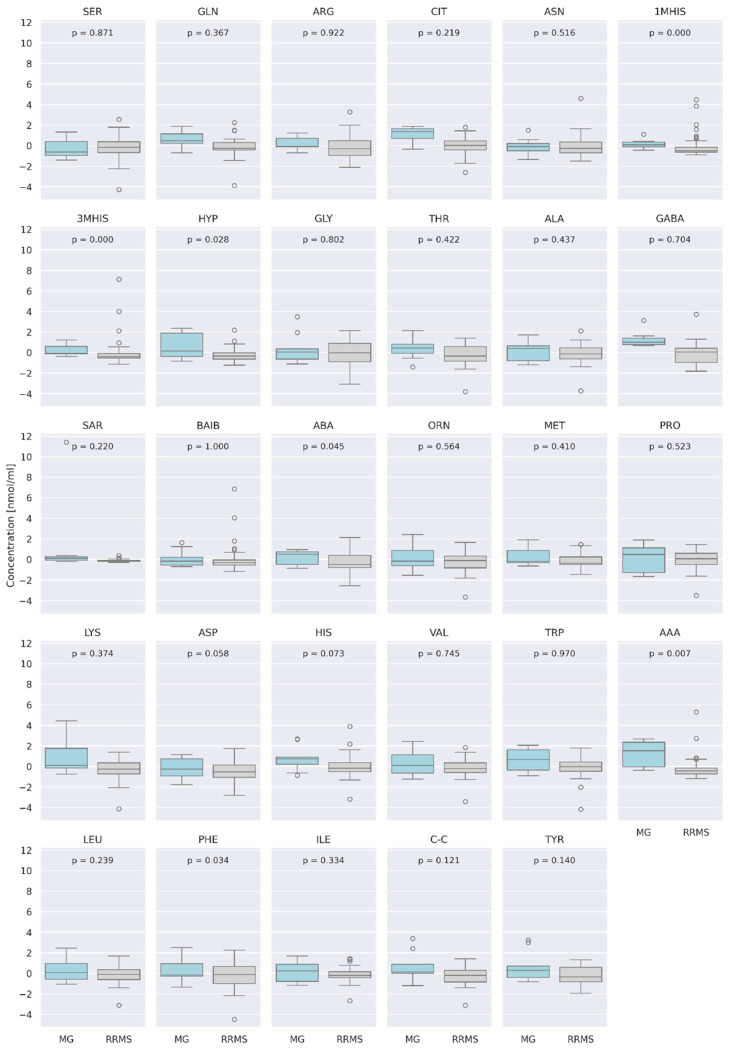
Centered and standardized AA concentrations between two groups, relapsing–remitting multiple sclerosis (RRMS—gray) and myasthenia gravis (MG—blue, represented as boxplots. The dots denote outlying observations.

At α = 0.05, we observed a group of AA concentrations to be significantly different between RRMS and MG patients (Figure 9), i.e., 1MHIS (*p* = 0.0003), 3MHIS (*p* = 0.00015), HYP (*p* = 0.027), AAA (*p* = 0.006), and PHE (*p* = 0.034). After Bonferroni correction (corrected *p*-value = 0.0017), 3MHIS remained significant. Median concentration of 3MHIS is higher in MG compared to that of RRMS patients (*p* = 0.00015).

**Figure 10:**
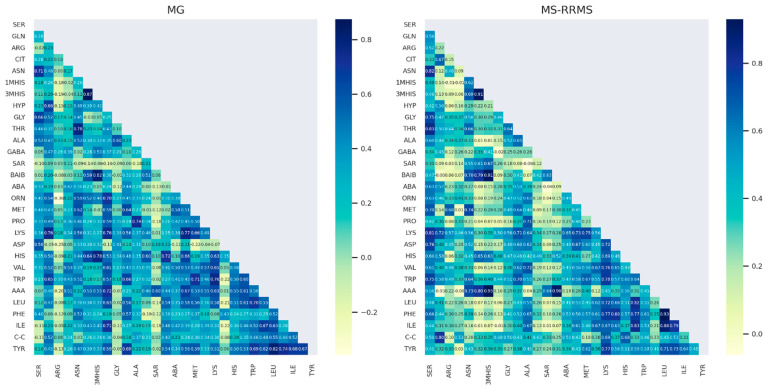
Correlation matrix demonstrating the degree of linearity in AA between the RRMS and MG patient groups. The number on the heatmaps denote R^2^.The lighter the color, the lower the correlation coefficient value between two AAs.

To further investigate the nature of the amino acids (AAs), we examined the correlations between them. Given that the distribution of each AA approximates a normal distribution and that they originate from similar pathways, a linear relationship between them is expected. Therefore, we used Pearson’s correlation to measure the linear dependence between variables. Below, we present a correlation matrix showing the degree of linear relationship between AAs in patients with MG and RRMS (Figure 10). Generally, the correlations are positive, with several AAs showing strong positive correlations with others (R^2^ > 0.8, dark blue), indicating that they essentially carry the same information. A visual inspection reveals that the correlation plots differ significantly between the two groups, with weaker linear correlations observed in the RRMS patient group.

## 4. Discussion

Although MS and MG are separate autoimmune diseases, some studies suggest that they coexist. The co-occurrence of MG and MS occurs more frequently than expected based on a chance association, and this association may be underdiagnosed due to the possible overlap of symptoms, especially bulbar and ocular manifestations, in which MG or MS may mimic each other, leading to an underestimation of the incidence combined occurrence of MG and MS [26]. In particular, two early symptoms of the disease—fatigue and diplopia—require caution in the proper diagnosis of the underlying disease. This aspect is important in the case of early MS symptoms in the complete absence or presence of only a single demyelinating lesion in the brain and/or spinal cord and existing contraindications to lumbar puncture. Moreover, with the increasing incidence of late-onset MG, patients may have foci of vascular damage in the central nervous system, some of which may be difficult to distinguish from demyelinating changes [6]. In such cases, patients are particularly vulnerable to misdiagnosis and having a peripheral marker that can distinguish MG from MS may be of significant support to clinicians.

Despite the differences in the pathologies of MS and MG, there are immunological similarities. Both MS and MG are considered largely T cell mediated [6]. Although the primary cause of MG development is autoantibodies against Ach receptors at the neuromuscular junction, the mechanism underlying the autoimmune response appears to be initiated by T cell activation. Alternatively, although multiple sclerosis is primarily mediated by T cells, there is some evidence that B cells and self-reactive antibodies also play a role in the pathogenesis of multiple sclerosis [26].

In T cells, AA are involved in many functions that include, but are not limited to, providing the building blocks of proteins, nucleotides, and lipids, regulating the epigenome, and maintaining redox balance, which are crucial for maintaining T cell function and differentiation capacity [27].

Comparing the patients (patients with MS and MG, n = 149) with the control group (n = 53), we found statistically significant differences in the concentrations of AAs, such as: ARG, CIT, M1HIS, ABA, PRO, TRP. Four of them, ARG, PRO, TRP, CIT, remained significant after taking multiple testing into account. Previous studies have indicated a differentiation between the control group of healthy people and patients with MG and MS due to the concentration of ARG [20,21,22].

AAs such as arginine and tryptophan seem particularly interesting due to their involvement in the regulation of the immune response [28]. Arginine, by participating in the synthesis of non-protein compounds such as nitric oxide or polyamines, plays an important role in vasodilation, calcium release, neurotransmission, and immune response. The research results indicate an important role of NO-dependent processes in both autoimmune diseases and chronic inflammation. Increasing the amount of NO in the body is associated with exacerbation of inflammation [29]. ARG catabolism occurs through multiple pathways, including degradation by iNOS to nitric oxide (NO) and CIT, as well as arginase-mediated degradation to produce PROs, urea, ornithine, and polyamines. Therefore, the higher serum ARG and PRO concentrations with lower CIT concentration found in our study may indicate a shift in ARG metabolism towards reduced degradation by iNOS [30].

When considering the comparison between AA concentrations in MS and MG patients we found three AAs that were significantly different in the MS and MG groups after correcting for multiple testing (CIT, GABA, and AAA). Higher concentrations of AAs that showed significant differences were observed in patients with myasthenia gravis. Based on the comparative analysis by gender, no significant differences were observed between women and men in the MS group. Previous studies did not show any differences in concentration between MG and MS patients and a control group of healthy people [20,22].

Knowledge about AAA function or the potential mechanisms linking AAA to the disease processes is limited. AAA is produced by the breakdown of the essential AA lysine and is metabolized mainly in the mitochondria. AAA is structurally similar to glutamate. In studies conducted on animal models, it was observed that AAA significantly reduces glutamate uptake. Furthermore, AAA induced the production of reactive oxygen and nitrogen species, lipid peroxidation, and protein oxidation, in addition to reducing antioxidant defense. It has been indicated that the disruption of glutamatergic neurotransmission and redox homeostasis by AAA may play a role in the neurological symptoms, among others, accompanying α-ketoadipic acidosis [31,32].

The obtained research results indicate reduced CIT concentration in MS patients compared to MG. The study by Rzepliński et al. indicated a reduced level of CIT in patients with MS compared to the control group, which was probably related to the disorder of Arg metabolism [20]. Reduced Arg levels in MS patients compared to MG may confirm this hypothesis. However, it is worth noting that another study hypothesized that demyelination in multiple sclerosis leads to increased release of citrulline from the brain into the circulation [33].

Most circulating citrulline is produced by the transformation of dietary glutamine and arginine by enterocytes of the intestinal epithelium. The released CIT is largely taken up by the kidneys, where almost 100% of it is metabolized by arginosuccinate synthetase and lyase to produce arginine [34]. In addition to the gut–renal axis and post-translational deamination, citrulline can be produced by the action of two other substances. First, citrulline acts as an intermediate in the urea cycle, where it is synthesized from ornithine and carbamoyl phosphate by ornithine carbamoyl transferase. Second, citrulline is a byproduct of NO production by nitric oxide synthase (NOS) from arginine [35].

Another AA that allowed for the statistically significant differentiation of the MS patient group from the MG patient group was gamma-aminobutyric acid (GABA). GABA is the main inhibitory neurotransmitter in the mammalian central nervous system and is directly responsible for the regulation of muscle tone in humans. GABA is also a modulator of the immune system [36]. The results obtained in our study indicate higher GABA concentrations in patients with MG compared to MS. Reduced GABA concentration in the CSF compared to the control group of healthy people has also been confirmed in other studies [21,37,38].

The model course of MS is an initial relapsing–remitting phase (RRMS), which, after a certain period, progresses to disability and transitions to a secondary progressive phase (SPMS). Due to diagnostic difficulties in differentiating myasthenia gravis and multiple sclerosis in their initial phase, we decided to isolate a group of patients with RRMS and myasthenia gravis to assess possible differences in the AA profiles of these groups. Both groups are similar in terms of age and sex distribution.

We reported that there were differences in the overall AA concentrations between the two disease subtypes (MG vs. RRMS); however, a lot of outlying observations were present in the patients’ data and the results should be treated with caution.

When considering the comparison between RRMS and myasthenia patients, we found differences in several AA concentrations (1MHIS, HYP, AAA, PHE), with 3MHIS being significantly higher in MG patients when correcting for multiple testing.

The increased 3MHIS concentration in MG patients compared to the healthy controls has been confirmed in previous studies [22]. It has been found that 3-methylhistidine (3MH) is formed as a result of the post-translational modification of histidine residues, which is an essential AA with a wide range of functions in the body. It is believed that the presence of free 3MH in the body is the result of the breakdown of myofibrillar proteins, therefore its possible use as a marker of myofibrillar protein catabolism is indicated [39,40]. An increase in the loss of muscle proteins is observed, among others, in neuromuscular diseases such as MG or Duchenne syndrome [41].

The visual inspection of AA concentration in a function of age between RRMS and MG patients showed a positive linear trend with age. No clear direction of linear trend was observed for AA concentrations in a function of duration (the same reported when duration was divided into two categories). A correlation plot visualizing the linear relationship between the AAs showed a vast majority of positive correlations in both groups of patients. Visually, both correlation plots are quite different, with RRMS patients characterized by weaker linear relationships between AA.

## 5. Conclusions

Multiple sclerosis and myasthenia gravis are autoimmune neurological diseases. Research indicates the possible involvement of AA in the modification of the immune response, which is a key mechanism in the initiation of autoimmune diseases.

In the presented work, the metabolic profiles of 29 AAs were determined using LC–MS/MS in order to capture specific changes in AA profiles between a group of patients with MG and MS.

Comparison of the AA concentrations between the groups showed statistically significant differences (α = 0.05) between GLN, CIT, GABA, SAR, His, AAA. After Bonferroni correction (corrected *p*-value = 0.0017), the concentrations of CIT, GABA, and AAA remained significant.

Statistical tests showed the existence of differences between disease types (RRMS, PPMS, SPMS, GMG) in terms of AA concentrations. RRMS and MG share many similarities, therefore a comparative analysis of AA profiles between these groups of patients was also performed. Bonferroni correction confirmed a significantly higher concentration of 3MHIS in MG compared to RRMS patients.

This study showed that AAs may be involved in the mechanisms underlying the pathogenesis of MG and MS diseases. The demonstrated differences may result from the participation of AA in immune responses, neurodegeneration processes, and construction of muscle proteins. The common pathogenesis of both diseases associated with an autoimmune background, involvement of T and B lymphocytes, and overlapping selected clinical symptoms may cause difficulties in the differential diagnosis of both diseases. At the same time, the identification of changes in AA metabolism in MS and MG can more precisely determine which of them are more likely to be attributed to autoimmune diseases of the neuromuscular junction and to the central nervous system. The obtained results may indicate AAs as potential biomarkers of autoimmune neurological diseases. The presented study has some limitations, including balancing the size of the study groups and the gender distribution in the group of patients with MG.

Further work is necessary, including a larger study group, limiting possible confounding factors, e.g., medications and diet, in order to correlate the results of previous studies with other possible autoimmune neurological diseases.

## Figures and Tables

**Table 1:** Demographic and clinical data of patients.

	MS	MG
Number of subjects (n)	121	28
Sex (male/female)	42/80 (34%/66%)	3/25 (10.7%/89.3%)
Age, years	52.5 ± 11.61 Min–Max: 23–77	48.92 ± 12.61 Min–Max: 29–75
Disease duration (years) [range]	16 ± 8.4	7.85 ± 6.53
Median EDSS score (IQR)	6 (4.0–6.5)	n.a.
MS type n (%)	RRMS: 41 (34%)	n.a.
	SPMS: 55 (45%)	
	PPMS: 25 (21%)	
MG type n (%)		GMG: 25 (89.3%)
		OMG: 3 (10.7%)
AChRAb		Yes: 21 (75%)
		No: 7 (25%)

MS—multiple sclerosis; MG—myasthenia gravis, RRMS—relapsing–remitting multiple sclerosis; SPMS—secondary progressive multiple sclerosis; PPMS—primary progressive multiple sclerosis; EDSS—Expanded Disability Status Scale; IQR—interquartile range; GMG—generalized myasthenia gravis; OMG—ocular myasthenia gravis, AChRAb—acetylcholine receptor antibody, n.a.—not applicable.

## Data Availability

Data are unavailable due to privacy or ethical restrictions.

## References

[1] DeMaio A., Mehrotra S., Sambamurti K., Husain S. (2022). The Role of the Adaptive Immune System and T Cell Dysfunction in Neurodegenerative Diseases. J. Neuroinflamm..

[2] Ha J.C., Richman D.P. (2015). Myasthenia Gravis and Related Disorders: Pathology and Molecular Pathogenesis. Biochim. Biophys. Acta (BBA)-Mol. Basis Dis..

[3] Ghasemi N., Razavi S., Nikzad E. (2017). Multiple Sclerosis: Pathogenesis, Symptoms, Diagnoses and Cell-Based Therapy. Cell J..

[4] Dong D., Chong M.K.C., Wu Y., Kaminski H., Cutter G., Xu X., Li H., Zhao C., Yin J., Yu S. (2020). Gender Differences in Quality of Life among Patients with Myasthenia Gravis in China. Health Qual. Life Outcomes.

[5] Harbo H.F., Gold R., Tintora M. (2013). Sex and Gender Issues in Multiple Sclerosis. Ther. Adv. Neurol. Disord..

[6] Danikowski K.M., Jayaraman S., Prabhakar B.S. (2017). Regulatory T Cells in Multiple Sclerosis and Myasthenia Gravis. J. Neuroinflamm..

[7] Kohler I., Verhoeven A., Derks R.J., Giera M. (2016). Analytical Pitfalls and Challenges in Clinical Metabolomics. Bioanalysis.

[8] Lu Y., Wang C., Chen Z., Zhao H., Chen J., Liu X., Kwan Y., Lin H., Ngai S. (2012). Serum Metabolomics for the Diagnosis and Classification of Myasthenia Gravis. Metabolomics.

[9] Blackmore D., Siddiqi Z., Li L., Wang N., Maksymowych W. (2019). Beyond the Antibodies: Serum Metabolomic Profiling of Myasthenia Gravis. Metabolomics.

[10] Rispoli M.G., Valentinuzzi S., De Luca G., Del Boccio P., Federici L., Di Ioia M., Digiovanni A., Grasso E.A., Pozzilli V., Villani A. (2021). Contribution of Metabolomics to Multiple Sclerosis Diagnosis, Prognosis and Treatment. Int. J. Mol. Sci..

[11] Ling Z.N., Jiang Y.F., Ru J.N., Lu J.H., Ding B., Wu J. (2023). Amino Acid Metabolism in Health and Disease. Signal Transduct. Target. Ther..

[12] Kurniawan H., Soriano-Baguet L., Brenner D. (2020). Regulatory T Cell Metabolism at the Intersection between Autoimmune Diseases and Cancer. Eur. J. Immunol..

[13] Antony I.R., Wong B.H.S., Kelleher D., Verma N.K. (2023). Maladaptive T-Cell Metabolic Fitness in Autoimmune Diseases. Cells.

[14] Socha E., Koba M., Kośliński P. (2019). Amino Acid Profiling as a Method of Discovering Biomarkers for Diagnosis of Neurodegenerative Diseases. Amino Acids.

[15] Corso G., Cristofano A., Sapere N., La Marca G., Angiolillo A., Vitale M., Fratangelo R., Lombardi T., Porcile C., Intrieri M. (2017). Serum Amino Acid Profiles in Normal Subjects and in Patients with or at Risk of Alzheimer Dementia. Dement. Geriatr. Cogn. Disord. Extra.

[16] Figura M., Kuśmierska K., Bucior E., Szlufik S., Koziorowski D., Jamrozik Z., Janik P. (2018). Serum Amino Acid Profile in Patients with Parkinson’s Disease. PLoS ONE.

[17] Kono M., Yoshida N., Tsokos G.C. (2021). Amino Acid Metabolism in Lupus. Front. Immunol..

[18] Lieu E.L., Nguyen T., Rhyne S., Kim J. (2020). Amino Acids in Cancer. Exp. Mol. Med..

[19] Negrotto L., Correale J. (2017). Amino Acid Catabolism in Multiple Sclerosis Affects Immune Homeostasis. J. Immunol..

[20] Rzepiński Ł., Kośliński P., Kowalewski M., Koba M., Maciejek Z. (2023). Serum Amino Acid Profiling in Differentiating Clinical Outcomes of Multiple Sclerosis. Neurol. I Neurochir. Pol..

[21] Pashaei S., Yarani R., Mohammadi P., Emami Aleagha M.S. (2022). The Potential Roles of Amino Acids and Their Major Derivatives in the Management of Multiple Sclerosis. Amino Acids.

[22] Kośliński P., Rzepiński Ł., Daghir-Wojtkowiak E., Koba M., Maciejek Z. (2023). Serum Amino Acid Profiles in Patients with Myasthenia Gravis. Amino Acids.

[23] Rzepiński Ł., Kośliński P., Gackowski M., Koba M., Maciejek Z. (2022). Amino Acid Levels as Potential Biomarkers of Multiple Sclerosis in Elderly Patients: Preliminary Report. J. Clin. Neurol..

[24] Van Rossum G., Drake F.L. (2009). Python 3 Reference Manual.

[25] Rzepiński Ł., Zawadka-Kunikowska M., Newton J.L., Zalewski P. (2021). Cardiac Autonomic Dysfunction in Myasthenia Gravis and Relapsing-Remitting Multiple Sclerosis—A Pilot Study. J. Clin. Med..

[26] Dehbashi S., Hamouda D., Shanina E. (2019). Co-Occurrence of Multiple Sclerosis and Myasthenia Gravis: A Case Report and Review of Immunological Theories. Mult. Scler. Relat. Disord..

[27] Hope H.C., Salmond R.J. (2021). The Role of Non-Essential Amino Acids in T Cell Function and Anti-Tumour Immunity. Arch. Immunol. Ther. Exp..

[28] Bröer S., Bröer A. (2017). Amino Acid Homeostasis and Signalling in Mammalian Cells and Organisms. Biochem. J..

[29] Sellebjerg F., Giovannoni G., Hand A., Madsen H.O., Jensen C.V., Garred P. (2002). Cerebrospinal Fluid Levels of Nitric Oxide Metabolites Predict Response to Methylprednisolone Treatment in Multiple Sclerosis and Optic Neuritis. J. Neuroimmunol..

[30] Virarkar M., Alappat L., Bradford P.G., Awad A.B. (2013). L-Arginine and Nitric Oxide in CNS Function and Neurodegenerative Diseases. Crit. Rev. Food Sci. Nutr..

[31] Desine S., Gabriel C.L., Smith H.M., Antonetti O.R., Wang C., Calcutt M.W., Doran A.C., Silver H.J., Nair S., Terry J.G. (2023). Association of Alpha-Aminoadipic Acid with Cardiometabolic Risk Factors in Healthy and High-Risk Individuals. Front. Endocrinol..

[32] da Silva J.C., Amaral A.U., Cecatto C., Wajner A., dos Santos Godoy K., Ribeiro R.T., de Mello Gonçalves A., Zanatta Â., da Rosa M.S., Loureiro S.O. (2017). α-Ketoadipic Acid and α-Aminoadipic Acid Cause Disturbance of Glutamatergic Neurotransmission and Induction of Oxidative Stress In Vitro in Brain of Adolescent Rats. Neurotox. Res..

[33] Vyver M.V., Beelen R., De Keyser J., Nagels G., Van Binst A.M., Verborgh C., D’haeseleer M. (2018). Plasma Citrulline Levels Are Increased in Patients with Multiple Sclerosis. J. Neurol. Sci..

[34] Crenn P., Coudray-Lucas C., Thuillier F., Cynober L., Messing B. (2000). Postabsorptive Plasma Citrulline Concentration Is a Marker of Absorptive Enterocyte Mass and Intestinal Failure in Humans. Gastroenterology.

[35] Breuillard C., Cynober L., Moinard C. (2015). Citrulline and Nitrogen Homeostasis: An Overview. Amino Acids.

[36] Watanabe M., Maernura K., Kanbara K., Tamayama T., Hayasaki H. (2002). GABA and GABA Receptors in the Central Nervous System and Other Organs. Int. Rev. Cytol..

[37] Gao F., Yin X., Edden R.A.E., Evans A.C., Xu J., Cao G., Li H., Li M., Zhao B., Wang J. (2018). Altered Hippocampal GABA and Glutamate Levels and Uncoupling from Functional Connectivity in Multiple Sclerosis. Hippocampus.

[38] Nantes J.C., Proulx S., Zhong J., Holmes S.A., Narayanan S., Brown R.A., Hoge R.D., Koski L. (2017). GABA and Glutamate Levels Correlate with MTR and Clinical Disability: Insights from Multiple Sclerosis. NeuroImage.

[39] Nagasawa T., Yoshizawa F., Nishizawa N. (1996). Plasma N-Methylhistidine Concentration Is a Sensitive Index of Myofibrillar Protein Degradation during Starvation in Rats. Biosci. Biotechnol. Biochem..

[40] Sjijlin J., Hjort G., Friman G., Hambraeus L. (1987). Urinary Excretion of L-Methylhistidine: A Qualitative Indicator of Exogenous 3-Methylhistidine and Intake of Meats From Various Sources. Metabolism.

[41] Mary P., Servais L., Vialle R. (2018). Neuromuscular Diseases: Diagnosis and Management. Orthop. Traumatol. Surg. Res..

